# Theoretical effectiveness of steam inhalation against SARS-CoV-2 infection: updates on clinical trials, mechanism of actions, and traditional approaches

**DOI:** 10.1016/j.heliyon.2022.e08816

**Published:** 2022-01-23

**Authors:** Md. Nafees Rahman Chowdhury, Yasin Arafat Alif, Safaet Alam, Nazim Uddin Emon, Fahmida Tasnim Richi, S. M. Neamul Kabir Zihad, Md. Tohidul Islam Taki, Mohammad A. Rashid

**Affiliations:** a Department of Pharmacy, Faculty of Pharmacy, University of Dhaka, Dhaka 1000, Bangladesh; b Department of Pharmacy, State University of Bangladesh, 77 Satmasjid Road, Dhanmondi, Dhaka 1205, Bangladesh; c Department of Pharmacy, Faculty of Science and Engineering, International Islamic University Chittagong, Chittagong 4318, Bangladesh; d Department of Pharmaceutical Chemistry, Faculty of Pharmacy, University of Dhaka, Dhaka 1000, Bangladesh

**Keywords:** SARS-CoV-2, COVID-19, Steam, Vapour, Inhalation therapy, Respiratory illness, Clinical study

## Abstract

Steam inhalation therapy can be a contemporary approach for COVID-19 affected patients of all age groups to manage respiratory conditions, though it presently lacks the scientific backing to establish itself as a befitting practice. The age of COVID-19 has facilitated this traditional home remedy to resurface among the general mass as a helpful approach for the prevention and adjuvant treatment of the disease. In this review, the means of SARS-CoV-2 infection and impact of the parameters, namely steam inhalation and heat on such infection has been delineated via enumerating the effect of the parameters in the human body and against SARS-CoV-2. The literature search was conducted using PubMed, Web of Science, Scopus, ScienceDirect, Wiley Online Library, Google Scholar, and CNKI Scholar databases. The keywords used in the survey include ‘Steam inhalation’, ‘SARS-CoV-2’, ‘COVID-19’, ‘Clinical study’, ‘Mechanism of action’, ‘Traditional uses’, ‘Phytochemistry’ and ‘Adverse effects’. Clinical studies concerning steam inhalation by COVID-19 patients have been comprehended to demarcate the scientific obscurity of the practice. The safety profile of the procedure has also been outlined emphasizing evading measures against COVID-19 and other related disease states. To recapitulate, application of the steam inhalation with herbal concoctions and phytochemicals having folkloric prevalence as an inhalable remedy against respiratory illnesses has been explored in this review work to focus on a new aspect in the COVID-19 treatment paradigm using steam and progress of further research hither.

## 1 Introduction

**Figure 1:**
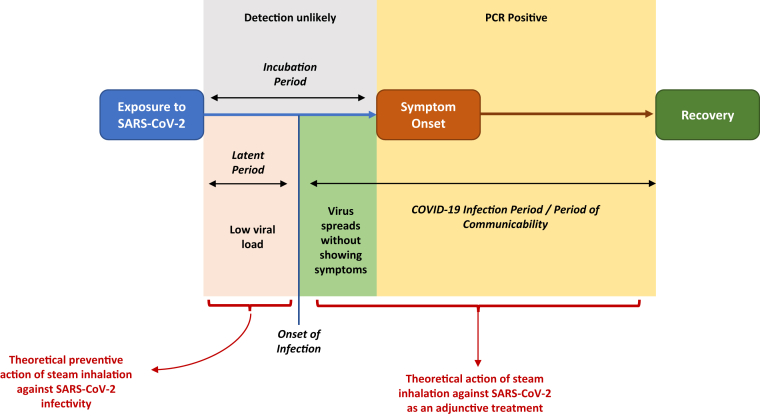
Theoretical effectiveness of steam inhalation in different periods of COVID-19 infection.

Severe acute respiratory syndrome coronavirus 2 (SARS-CoV-2) is an RNA virus that is the causative agent of coronavirus disease 2019 (COVID-19). This is primarily characterized by a respiratory illness which has already ensued a global spreading and facilitated the disease being designated as a pandemic by The World Health Organization (WHO) on March 11, 2020 (Al Naggar et al., 2021; Di Gennaro et al., 2020; Tang et al., 2020). Parts of the upper respiratory tract, namely nasal mucosa, larynx, and pharynx are the prominent sites for the initial phase of the infection (Wölfel et al., 2020). This corresponds to the beginning episode of the 3–7 days long latency period of the virus (Figure 1) (Kayode et al., 2021; Xiang et al., 2021) after entering the body through the nose, mouth or eyes (Di Gennaro et al., 2020; Swain and Agrawala, 2020). Consequently, the lower respiratory system is pervaded by the virions. Afterward, the SARS-CoV-2 spike glycoprotein binds with angiotensin-converting enzyme 2 (ACE2) of the lung epithelium, enabling further aggravation of infection (Daley et al., 2020; Elebeedy et al., 2021). The earliest binding interaction of ACE2, the entry protein of SARS-CoV-2, with the external glycoprotein structure occurs in the nasal epithelium wherein the nasal epithelium reportedly expresses more of the entry protein than the principal entry site of lung epithelium itself (Bleier et al., 2021; la Marca et al., 2021). As such, the spike glycoprotein of SARS-CoV-2, central to viral access to the host cells and replication resulting in disease progression, is a prospective target for preventive and curative interventions to halt viral proliferation in the airways (la Marca et al., 2021).

Steam inhalation therapy is an eminent traditional approach, typically in the home setting. It is practiced as an adjuvant treatment paradigm to heal infections associated with the respiratory system such as the rhinovirus-mediated common cold, influenza virus-mediated common flu as well as croup and bronchiolitis among others. As such, steam inhalation can be recognized as an intuitive outlook to incapacitate SARS-CoV-2 infection at the very preliminary stage (Swain and Sahu, 2021; Uy et al., 2020). This is so because, firstly, the structure of the influenza virus exhibits closeness with that of the SARS-CoV-2, and secondly, following the earlier reports of rhinovirus being weakened from resuming replication and hence, having curtailed replication with the *in vitro* application of heat at 33–43 °C (Conti et al., 1999). In addition, the influenza virus, also reported to have a heat correlation with SARS-CoV-2 has been inactivated *in vivo* and *in vitro* at a temperature more than 30 °C (Lowen and Steel, 2014). Studies have found that in liquid conditions, SARS-CoV-2 spike glycoprotein is irreversibly denatured after heating for 30 min at 56 °C with subsequent loss of pathogenicity while the incubation of the virus at 70 °C plummeted inactivation time to a duration of 5 min from 14 days (Chin and Poon, 2020). Additionally, according to another study, full inactivation of the virus is not achieved by heating to 56 °C or 60 °C for up to 60 min. Rather, heating to 80 °C for 90 min or 95 °C for 1 or 5 min can obtain full inactivation along with minimized RT-PCR sensitivity through increased cycle threshold (ct) values, indicating lowered viral load (Burton et al., 2021). Moreover, the heat inactivation of coronaviruses at the sub-second level, namely the complete inactivation of the Murine coronavirus (MHC) model at 83.4 °C in 1.03 s potentially consolidates the theorized effect of steam and heat against SARS-CoV-2 (Jiang et al., 2021).

A survey work conducted in 2016, revealed that 80% of the general practitioners advise their patients to avail steam inhalation therapy (Al Himdani et al., 2016). Although the nasal route of inhalation remains persistent (Akhavani and Baker, 2005), the method of generating and intaking steam varies from traditional as well as popular ones like bending over a bowl containing hot or boiling water with a cloth overhead, taking a hot bath, staying in steam rooms, hammams, and saunas to utilizing modern equipment like electrical steamers and nasal cannulae (Cohen, 2020; Uy et al., 2020). The traditional and ostensibly, the most common procedures of steam inhalation furnishes a temperature around 70–80 °C which commensurates well above the temperature necessitating the instability and loss of infectivity of SARS-CoV-2 (Pawar et al., 2020). However, such action can only be expected as long as and up to the virions are present in the upper respiratory tract, partly analogous to the pre-infective period since steam cannot reach the lower airways (la Marca et al., 2021). On such grounds, this study projects to compile salient details on the mechanism of action, clinical reports, traditional practice-based evidence, phytochemical studies and adverse effects of steam inhalation therapy for averting as well as adjunctively treating COVID-19 to develop a consolidated insight into the traditional practice.

## 2 Article search strategy

**Figure 2:**
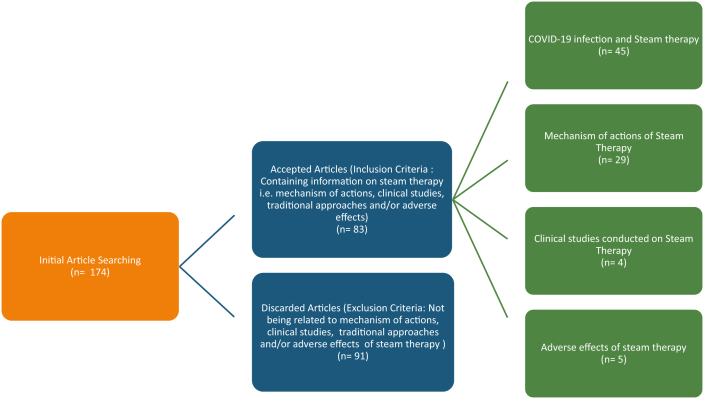
Flowchart for article search, screening and selection in literature review.

To summarize the findings regarding the candidacy of steam inhalation against SARS-CoV-2 infection, a thorough literature search was conducted using PubMed, Web of Science, Scopus, ScienceDirect, Wiley Online Library, Google Scholar, and CNKI Scholar databases. The keywords used in the survey include ‘Steam inhalation’, ‘SARS-CoV-2’, ‘COVID-19’, ‘Clinical study’, ‘Mechanism of action’, ‘Traditional uses’, ‘Phytochemistry’ and ‘Adverse effects’. The acceptance criteria for peer-reviewed scientific journals were specified to information on steam therapy i.e. mechanism of actions, clinical studies, traditional approaches and/or adverse effects only. Out of 153 identified papers found, 83 distinctive articles met the inclusion criteria according to the evaluation of the reviewers and were incorporated and reported in this comprehensive review (Figure 2).

## 3 Mechanism of actions

### 3.1 Mechanism of action of steam in the body

Heat, the quintessential facet of steam, has a significant impact on host defense, physiological immunity, viral load, virulence and activates adaptive thermoregulatory mechanisms that can raise or decrease body temperature to restore homeostasis (Schieber and Ayres, 2016). Moreover, the temperature increase of the entire body further enhances the second line of defence by inducing heat stress that imitates fever symptoms (Schieber and Ayres, 2016). Environment, source, temperature, humidity, area, and the time limit of heat application play vital roles in the mechanism to prevail over viral infection (Schieber and Ayres, 2016). As such, heat stress and hyperthermia have several effects that can prevent viral infections. Pathogens are directly inhibited, the immune system is enhanced (both innate and adaptive), and regulatory mechanisms that reduce inflammatory responses and avoid unrestricted tissue damage are activated (Evans et al., 2015).

### 3.2 Mechanism of action of steam on respiratory and cardiovascular system

**Figure 3:**
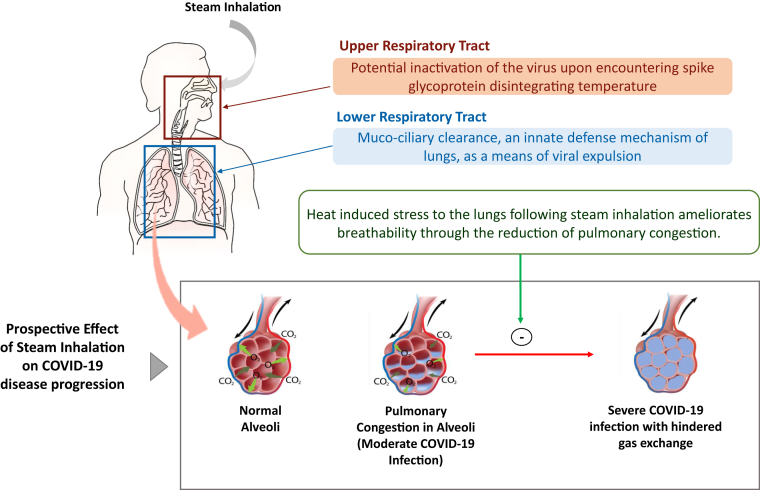
Mechanism of action of steam induced heat on the respiratory tract (upper and lower) and its potential corresponding effect on COVID-19 disease progression.

Inhalation of steam creates a stressed situation for the lungs. Due to this heat-induced stress, the lungs' breathability is enhanced by reducing pulmonary congestion and increment of tidal volume, vital capacity, airflow, and the forced expiratory lungs volume (Laitinen et al., 1988). Inhalation of hot air strengthens the first line of defence of the immune system by directly inhibiting or disabling virions in the initial airways of the body where they first invade and on top of that, facilitate muco-ciliary clearance, which can be further improved by steam inhalation (Figure 3) (Gujrathi et al., 2016).

**Figure 4:**
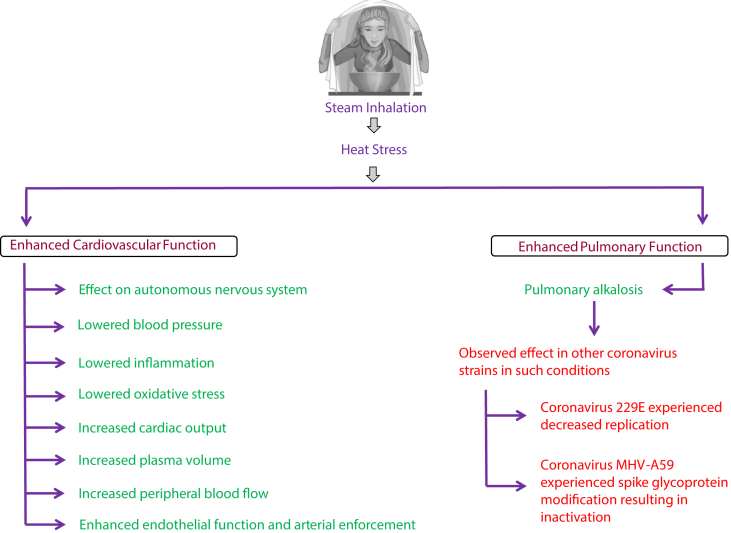
Mechanism of cardiac and pulmonary action of steam induced heat and observed effects in other strains of coronavirus.

Steam inhalation induced heat-stress enhances cardiovascular function by affecting the autonomous nervous system, lowering blood pressure, inflammation, and oxidative stress, increasing cardiac output, plasma volume, and peripheral blood flow, and enhancing endothelial function, lipid profile, and arterial enforcement (Heinonen and Laukkanen, 2018; Kunutsor et al., 2018; Laukkanen and Kunutsor, 2019; Laukkanen et al., 2018). Alteration of blood pH through hyperventilation caused by hyperthermia, followed by pulmonary alkalosis, is another benefit of heat stress (Tsuji et al., 2016). Therefore, strains such as coronavirus 229E, which has shown utmost pathogenicity in acidic conditions, can be mobilized and their replication can be slowed down or stopped in this induced alkaline environment (Lamarre and Talbot, 1989). At a pH of 8, at 37 °C, the coronavirus MHV-A59 undergoes conformational modifications in the spike glycoprotein, resulting in fast and permanent inactivation and loss of infectivity (Figure 4) (Sturman et al., 1990).

### 3.3 Cellular response to heat stress

Pyretic temperatures trigger numerous cellular responses, including a complex reciprocal modulation of the heat shock response pathway, inflammation and immune system activation (Singh and Hasday, 2013). Heat shock proteins (HSPs) are released in the lipid bilayer membrane of cells due to the stimulation of heat stress (Iguchi et al., 2012). Its main function is to serve as a chaperone and to prevent thermal disruption to immune cells and proteins. It also has its fair share of effort in activation of lymphocytes and macrophages, and antigen and cross-presentation, furthermore in activation and maturation of dendritic cells (Singh and Hasday, 2013; Tsan and Gao, 2009). Acute heat stress amplifies NK cell activity (interleukin-2 originated). T-lymphocytes produce 10 times more interferon than normal and even elevate the TNF-alpha response of monocytes (Downing et al., 1988; Kappel et al., 1991; Zellner et al., 2002). On the contrary, constant heat stress results in an increment of cytotoxicity of NK cells, reduction of cortisol and adrenaline, and magnification of proliferative response of B cells (Tomiyama et al., 2015).

Heat-stress induced by steam inhalation and other processes enhances the immune system's stimulation, which can be elevated by further exposure to cold (Heinonen and Laukkanen, 2018). Elevation in NK cell count and activity and circulating levels of IL-6 and leucocytosis and granulocytosis is observed due to this further cold exposure after heat stress (Brenner et al., 1999). Additionally, this process helps detoxify as diuresis occurs due to blood being shunted in the viscera (Cochrane, 2004; Epstein, 1978).

### 3.4 Mechanism of actions of steam against SARS-CoV-2

**Figure 5:**
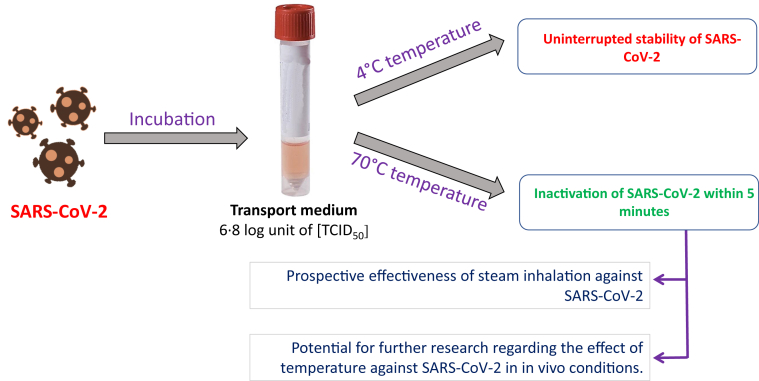
Experiment conducted to determine the thermolability of SARS-CoV-2.

As per an experiment conducted by the School of Public Health, LKS Faculty of Medicine of The University of Hong Kong, it has been observed that heat at different studied temperatures can cause the inactivation of SARS-CoV-2 *in vitro* (Chin and Poon, 2020). An *in vitro* study conducted to investigate the influence of temperature on SARS-CoV-2 stability through incubating it in a transport medium at the concentration of ∼6 · 8 log unit of [TCID_50_] (50% tissue culture infectious dose) per mL for 14 days found that the virus exhibited high stability around 4 °C while heat sensitivity at higher temperature was also distinguished. Accordingly, at 4 °C, only 0.7 log-unit diminutions of infectious viral titer were observed on the 14^th^ day, whereas at 70 °C, viral inactivation time came down to 5 min (Figure 5) (Chin and Poon, 2020). According to Lowff's model, the proliferation of rhinoviruses can be restricted in the respiratory tract mucosa by increasing the mucosal temperature to 43 °C for 3 sessions enduring 30 min with a break of 2 h between each session (Yerushalmi et al., 1982; Yerushalmi and Lwoff, 1980). Thus, owing to the thermolability of SARS-CoV-2, it has been hypothesized that steam inhalation therapy at 55–65 °C, if implemented shortly after contamination, could feasibly minimize the possibility of severe infection development by abating infection in the mucosa of upper airways (la Marca et al., 2021). Hence, the systematic application of the therapy was suggestive since contamination mostly occurs unsuspectingly (Janik et al., 2021). However, till now, no study reveals the temperature, humidity, and time necessary to cause the specific deactivation of SAR-CoV-2 *in vivo* (Cohen, 2020).

## 4 Clinical studies

Steam inhalation has been known for a long time regarding its efficacy against respiratory tract infections (Uy et al., 2020). It has been used as a conventional medical practice in households to treat respiratory diseases such as bronchitis, common viral colds, influenza, etc (Swain and Sahu, 2021). As per previous studies, influenza and SARS coronavirus show a correlation upon applying heat (Chan et al., 2011; Lowen and Steel, 2014). As SARS-CoV-2 is structurally similar to these two viruses, studies have been done to evaluate if steam inhalation can be a worthwhile practice against coronavirus (Pawar et al., 2020).

In July 2020, a group of researchers in Seven Hills Hospital of Mumbai, India aimed to determine the efficacy of steam inhalation as an adjunctive treatment against SARS-CoV-2. A group of asymptomatic and another group of mildly and moderately symptomatic patients and healthcare workers (doctors and nurses) were gathered and divided into 2 groups correspondingly. Group 1 consisted 25 healthcare workers and group 2 consisted 80 patients and healthcare workers. The first group involving asymptomatic patients was advised to inhale the steam for 5 min 2 times a day and the latter group was recommended to inhale the steam for 5 min every 3 h. The steam generally was around about 70 °C–80 °C. None of the first groups showed any symptoms in check-ups differing from 2 weeks to 2 months. In the mildly symptomatic patients of the second group, symptoms reverted by 3 days of steam inhalation and in the moderately symptomatic subgroup, 7–10 days of steam inhalation were required for the symptoms to revert. Most of the subjects (65) had a negative result in the Covid-19 test within 10 days of steam exposure, while the other 15 had negative consequences within 18 days (Pawar et al., 2020).

Later that year, another group of Italian researchers gathered 10 adult healthcare personnel from Meyer Children's University Hospital, Florence, who were affected by SARS-CoV-2. Then, a study was conducted to understand the role of thermal discharge of coronavirus via steam inhalation. The group was exposed to 55–65 °C temperature humidified steam for at least 20 min via 4 cycles of 5 min or 5 cycles of 4 min in an hour. The target was to measure viral shedding and viral elimination via CT values from RT-PCR testing after 4 days of following protocol. 7 out of the 10 patients could complete the protocol. The other 3 had various complications because they could not complete the protocol and their outcome has been treated independently. The results were encouraging as all 7 patients who completed the protocol accordingly tested negative following a swab 10 days later. The ones who didn't follow the complete protocol also had positive impacts (la Marca et al., 2021).

In 2021, research continued as another group of researchers India gathered 2 groups of symptomatic and asymptomatic healthcare professionals with an age range of 22–68 years and an average age of 38 ± 16 years. There were 44 (28 Male, 16 Female) and 52 (34 Male, 18 Female) individuals respectively in the mentioned groups and they were all asked to inhale 42 °C to 45 °C steam as an adjunctive treatment and advised to do check-up visits at week-1 and week-2 followed by the 1^st^ month and 2^nd^-month visit. The asymptomatic group was instructed to follow the procedure 3 times a day for 2 weeks. The symptomatic group was advised to follow it every 3 h for 2 weeks. Moreover, the CO-RADS score and degree of participation from the CT-scan of the subjects' thorax were also looked on. Only 4 out of the 52 asymptomatic patients showed mild symptoms in the first weeks' check-up and in the second week, the RT-PCR test result for this group of patients became negative. In the 44 patients of the symptomatic group, 36 had mild symptoms who were symptomless through 5 days of inhaling steam and 8 had moderate symptoms who became asymptomatic in 7 days of steam inhalation excluding only 1. This excluded patient had suffered from pneumonia which later turned into orotracheal intubation and he required COVID intensive care. 36 out of the 44 of this group gave a negative result in the RT-PCR test by 14 days and the remaining 8 followed them with negative consequences by 20 days. The degree of involvement for all the subjects of the symptomless group had a score from 0 to 5 (out of 20; which suggests minimal invasion) before steam inhalation and after following the protocol, all of their scores came down to 0 (no invasion). From the symptomatic group, 35 of the subjects CT scan was gathered of which 23 had a score of 0–5, 7 had a score of 6–10 (mild invasion) and 5 had a score of 11–15 (moderate invasion) before the protocol was followed and after the procedure was followed, only 2 had 0 to 5 score, and a patient each in 6–10 score and 11 to 15 score category (Swain and Sahu, 2021).

A 43 participant's case-control study was conducted at the Corona unit of Sher-e-Bangla Medical college hospital all of whom tested positive for the RT-PCR test (Sarker et al., 2021). To compare the effects of steam inhalation with medication to steam inhalation without medication, researchers conducted this study. The participants were divided into two groups: 16 people were designated as participants of control group who were exposed to plain water vapour as adjunctive treatment and rest of the 27 people were mentioned as participants of the study group inhaled vapour comprising Menthol 0.02 percent, Methyl salicylate 0.05 percent, N- Acetyl cysteine 1.2 gm percent, and Diclofenac sodium 1 gm percent twice a day as adjunctive treatment in addition with the standard treatment. The standard treatment was mentioned to be Diclofenac sodium 1 gm, N-acetyl cysteine 1.2 gm, menthol 20 mg, and methyl salicylic acid 50 mg are all contained in 100 gm of emulsion. The age range of the participants was from 20 years to above 75 years with most patients being in between 42 years and 52 years in both of the groups. In contrast with the control group, the study group had oxygen saturation levels increased 384.61 percent more in the morning and 515.79 percent more at night after regular inhalation of steam with the aforementioned drugs. Furthermore, patients in the study group spend roughly one day less in the hospital than those in the control group (Sarker et al., 2021).

## 5 Steam inhalation in local uses and traditional medicines

According to the World Health Organization, about 80% of the world's population living in developing countries extensively use traditional drugs in their communities (World Health Organization, 2000). The WHO has sorted out a catalogue of nearly 21,000 potentially medicinal plants worldwide, including around 2500 varieties from India alone (Seth and Sharma, 2004). The Government of India has identified various herbs used in conventional in-house inhalation therapy that have been seen to have therapeutic benefits in strengthening respiratory tracts and immune systems (Vellingiri et al., 2020). Steam inhalation with various combinations of herbal products could help stop the coronavirus, especially for the treatment of patients with not very serious symptoms (Marwah and Marwah, 2020).

### 5.1 Steam inhalation in ayurveda

Some Ayurvedic practitioners have also suggested adding mint leaves, and seeds of caraway with the steam inhalation procedure known to be potent against lung diseases (Golechha, 2020; Rajkumar, 2020). As an established procedure against upper respiratory tract problems such as bronchospasm, inflammation in the throat, and sinusitis, this has been a prime contender for home remedies for COVID-19 patients (Sathya et al., 2020; Tillu et al., 2020). Although no proven psycho-neuroimmune function is available, the chances are that this may change internal immune-inflammatory functions, and also by affecting mesolimbic and mesocortical neural connection, this enhanced steam inhalation can take the edge off stress, anxiety, and mood (Oken, 2008; Shisode, 2021).

### 5.2 Soap-steaming

A group of researchers has proposed soap steaming to remove coronavirus and any sort of virus from the upper respiratory system and early parts of the lungs (Marwah and Marwah, 2020). The procedure of soap steaming should be like the following: 100 g of cumin seed (powdered), 50 g of carom seed, 175 g of sodium chloride, 15 g of sodium stearate, and 1 ml eucalyptus oil containing 10 ml of coconut oil are blended and processed rigorously in and sealed in an airtight jar. Then 10 g of this blend can be applied either by a steam inhaler or by using around a litre of boiling water, and the steam should be inbreathed for 5–10 min through the nose and mouth. The research group believed that cuminaldehyde, a natural organic compound found in cumin and eucalyptus (Chalchat et al., 2001; Morshedi et al., 2015), can be effective in coronavirus inactivation as it has a chance to react with surface protein's amine groups (-NH_2_) (Marwah and Marwah, 2020). The virus's outer membrane can disintegrate through the very little amount of stearate and essential oils used in the blend along with the high temperature, which will result in inevitable incapacitation of the virus in the lungs and cleansing of the upper respiratory airway. Cumin and carom seed's volatile constituents, which include cuminaldehyde, terpenes, thymol, and others, are also relaxing to throats and lungs. Essential oils demonstrate preventive capability against various viruses but cannot hinder their entrance into the host body (Piątkowska and Rusiecka-Ziółkowska, 2016). It was suggested that the infected patients should use this method thrice a day (under supervision) and others can use this maybe once a day, following the protocol twice a week. The researchers also suggested more applications of lipids such as sodium stearate inhalers for hospitalized patients to intensify the results (Marwah and Marwah, 2020).

### 5.3 Steam inhalation with beehive products

In another study regarding the use of ancient procedures against COVID-19, researchers hypothesized that using beehive contents such as honey (aerosolized) and propolis tincture in steam inhalation procedures can be useful to reduce COVID-19 symptoms (Al Naggar et al., 2021). Products like Manuka honey in different thickness levels have provided, somewhat surprisingly, results such as the release of chemokines, cytokines, and enzymes that break down matrix (Minden-Birkenmaier et al., 2019). The lower level of oxygen, which is a very common occurrence in COVID-19 patients, can be resolved by using propolis to induce higher productivity from mitochondria in alveolar cells (Farooqui and Farooqui, 2012). Honey is a proven symptom reducer of tracheal and lung infections (Abuelgasim et al., 2021, Lauer et al., 2020). It energizes ciliary cells of the upper respiratory tract that move mucus in and out of the lungs, thereby making it runnier and thus aiding expectoration (Bustamante-Marin and Ostrowski, 2017). Recent studies have found that 14 selected terpenes and phenolic compounds found in honey and propolis have an overwhelming affinity towards 2 enzymes present in the novel coronavirus (2019-nCoV), the main protease (Mpro and RNA dependent RNA polymerase (RdRp) enzyme (Shaldam et al., 2021). Out of these compounds, *p-*coumaric acid, ellagic acid, kaemferol, and quercetin have a considerable opportunity to be used as an inhibitor to the virus as they have suggestively strong interaction with the enzymes mentioned above (Shaldam et al., 2021).

### 5.4 Kujifukiza

“*Kujifukiza*” is a Tanzanian traditional herbal medication where plant leaves are boiled in water and people gather around to inhale the volatile medicinal products (Kamazima et al., 2020). The main purpose here is not necessarily steam inhalation but inhalation of volatile medicinal oils. The oils show different activities towards viruses, bacteria and provide a soothing effect. However, it has no proven efficacy against COVID-19 (Kamazima et al., 2020). Still, the Tanzanian government urged the people to follow this ancient procedure to fight against this deadly virus there (Kamazima et al., 2020). Furthermore, 70 traditional medicinal experts from all over Africa had a virtual meeting with WHO in May 2020 and proposed to conduct clinical trials on all conventional medicine on the continent as they believe that the cure for COVID-19 could be right there (Kamazima et al., 2020).

## 6 Adverse effects

As the COVID-19 situation gets serious, a home remedy such as steam inhalation becomes more popular among common people. But it has its dangers too if the procedure is not done carefully, especially with children (Chiriboga et al., 2020). A survey from Children's Burns Trust in the UK suggests that more than 100 children suffering from burn injuries required emergency intervention every day, and the most common of these injuries were scald injuries. Since the early days of Covid, the number of scald burns cases has increased 30-fold in Birmingham Children's Hospital, resulting mostly from steam inhalation (Chiriboga et al., 2020).

Scald burns resulting from inhalation of steam surged following the emergence of the pandemic, as per another study in the Department of Burns and Plastic Surgery, SVP Institute of Medical Sciences and Research, Ahmedabad, Gujarat, India (YB et al., 2020). The major cause of such injury was the accidental toppling of the bowl containing hot boiled water used for steaming. Face, torso, upper and lower limbs and perineum were the common sites encountering the burn (YB et al., 2020). It has been also suggested that modern pieces of equipment like an electrical steamer, rhino-therm, and respirator could assure better safety in terms of inhaling steam alongside avoiding burns (YB et al., 2020).

Steam or moist air is nearly 4000 fold more capable to carry and release heat compared to dry air at any given temperature. Hence, inhaling moist hot air is substantially more injurious than inhaling dry air of equal temperature (Bhootra and Kitinya, 2005). It has been observed that the airway mucosa is susceptible to the impact of vapours of superheated steam at around 130 °C (Balakrishnan et al., 1996). Superheated steam inhalation can cause thermal damage to the lower respiratory tract, pulmonary insufficiency, bronchial mucosa damage, thermal tracheitis, fatal obstructive oedema of the glottis, haemorrhagic oedema of alveoli leading to hypoxia and anoxia (Still et al., 2001). In rare instances, massive laryngeal oedema can occur which may lead to asphyxia and death (Bhootra and Kitinya, 2005).

## 7 Discussion

Currently, the international crisis caused by COVID-19 has become more alarming with the raid of its fourth and fifth wave to many countries of the world (Kim et al., 2021; Sarhan et al., 2021). In such a situation, even the modern healthcare system and healthcare providers are being pestered with such hospital admission rush and to provide proper management and treatment of this emerging disease (Jean et al., 2020; Tillu et al., 2020). Several anti-COVID-19 drugs (Jean et al., 2020) and effective vaccines have been developed already (Alam et al., 2021a; Al-Karmalawy et al., 2021; Shehata et al., 2021). Even with the vaccines that have been found to be useful, the health sector is still facing difficulties to vaccinate all the people around the globe (Irwin and Nkengasong, 2021). Thus, alternative and complementary therapies can play a pivotal role in this emergency situation (Alam et al., 2021b). The main contentious issue has come up to their safety and efficacy due to the frequent mutation of SARS-CoV-2 virus strain (Al Naggar et al., 2021; Swain and Sahu, 2021). Because of several such shortfalls, we focused on preventive measures primarily prior to the curative approaches to combat the surge of COVID-19 (Tillu et al., 2020). Among all the approaches, steam inhalation is one of the preventive motions which are being used as adjuvant nursing care against COVID-19 infection, as it showed evident effectiveness in the case of both influenza and coronavirus earlier (Swain and Sahu, 2021). Moreover, steam inhalation has been used from ancient times to boost up one's immunity with less immunologic and inflammatory responses (Cohen, 2020; Vathanophas et al., 2019).

Furthermore, it has been shown to have promising actions against SARS-CoV-2 (Cohen, 2020) by boosting the body's natural immunity, amplifying the cardiac and pulmonary functions as delineated in the preceding sections. The propitious activity of steam inhalation has also been reported in several clinical studies (Pawar et al., 2020). Steam inhalation with aromatic oils (Tillu et al., 2020), several medical plant parts (Purohit, 2021), beehive products may also give positive outcomes against COVID-19. Therefore, steam inhalation can be an effective and convenient choice of options to combat SARS-CoV-2, supporting the healthcare system, as it is an easily adaptable, widespread, and economical method (Cohen, 2020).

## 8 Conclusion

Steam inhalation therapy appears to be a readily accessible, self-manageable and inexpensive approach across the globe considering its vast usage. It has been persistently observed that the administration of steam inhalation reduced clinical symptoms in infected patients though large-scale randomized controlled trials are still necessary to clarify and consolidate the effects as steam inhalation is still in a theoretical stage against COVID-19 infection. However, there were reports of adverse effects, namely accidental scald burns due to lack of care and especially supervision in paediatric patients while taking steam. In addition, inhaling the vapour of superheated steam at very high temperature (around 130 °C) can cause serious respiratory injury. It thus manifests that the therapy is only safe at the cost of maintaining strict caution and intensive supervision.

## Declarations

### Author contribution statement

All authors listed have significantly contributed to the development and the writing of this article.

### Funding statement

This research did not receive any specific grant from funding agencies in the public, commercial, or not-for-profit sectors.

### Data availability statement

Data included in article/supplementary material/referenced in article.

### Declaration of interests statement

The authors declare no conflict of interest.

### Additional information

No additional information is available for this paper.
